# Polyethylene glycol/silica and carbon black/silica xerogel composites as an adsorbent for CO_2_ capture

**DOI:** 10.3906/kim-2101-45

**Published:** 2021-10-17

**Authors:** Gülcihan GÜZEL KAYA

**Affiliations:** Department of Chemical Engineering, Faculty of Engineering and Natural Sciences, Konya Technical University, Konya, Turkey

**Keywords:** Silica xerogel composite, polyethylene glycol, carbon black, CO_2_ capture

## Abstract

Mesoporous polyethylene glycol (PEG)/silica and carbon black (CB)/silica xerogel composites were prepared by the sol-gel method as an adsorbent for CO_2_ adsorption. The CO_2_ adsorption process was carried out under pure CO_2_ atmosphere at room temperature in addition to ambient air. The xerogel composites with high surface area and pore volume showed better CO_2_ adsorption capacity than the pure silica xerogel. After modifying samples with propylene diamine using the wet impregnation method, an increase in CO_2_ adsorption capacity was observed for the samples except CB/silica xerogel composite. The highest CO_2_ adsorption capacity was determined as approximately 0.80 mmol/g for amine modified PEG/silica xerogel composite under pure CO_2_ exposure. According to the adsorption-desorption cyclic stability test, it was clear that the stable samples were obtained, which is a desirable property for all CO_2_ adsorbents. The promising findings revealed that the xerogel composites can be efficiently used as a CO_2_ adsorbent instead of conventional materials in many CO_2_ adsorption applications. Additionally, it can be expected that the xerogel composites can provide an effective adsorption process without high-cost, complexity, corrosion, and toxicity problems.

## 1. Introduction

An increase in the concentration of greenhouse gases resulted in global warming constitutes a serious problem all over the world [1]. Carbon dioxide (CO_2_), which contributes approximately 60% to global warming, is one of the main greenhouse gases generally originated from power plant fumes, vehicles, cement factories, steel industry, and human activities [2,3]. CO_2_ emission is expected to increase with rapid population growth and excessive energy demand [4]. International Panel on Climate Change (IPCC) estimates that the concentration of CO_2_ may reach 570 ppmv that leads adverse climate change and many irreversible human health problems if no precautions are taken in time [5,6]. So, many novel and useful CO_2_ capture processes such as amine, aqua ammonia and dual alkali absorption, membrane separation, cryogenic distillation, and adsorption have been developed by researchers in recent years [7,8]. In the adsorption processes, different types of solid adsorbents including metal-organic frameworks (MOFs) [9], covalent organic frameworks (COFs), covalent organic polymers (COPs) [10], activated carbon, zeolites [11], and silica-based materials [12] have been commonly utilized with high adsorption efficiency.

Silica-based materials with the advantages of ultralow density, high surface area, desired porosity, dielectric constant, transport properties, and stability are easily synthesized by the sol-gel method [13,14]. Depending on the drying method, the materials are denominated as xerogel (ambient pressure drying), aerogel (supercritical drying), and cryogel (freeze-drying) [15,16]. Silica xerogels are well known as promising materials with unique properties in various fields such as catalysis, adsorption, optical sensor production, and so on [17]. Silica xerogels are utilized for CO_2_ capture in spite of their low adsorption capacity compared to that of the other silica-based materials [18]. However, the adsorption capacity of these materials can be enhanced with amine impregnation or grafting [19] and incorporation of various components to the structure such as Al_2_O_3_, MgO, TiO_2_, larnite, wollastonite, carbon nanofiber, and so on [20–23].

There are a limited number of studies related to silica xerogels as an adsorbent for CO_2_ capture in the literature. For instance, Huang et al. [24] prepared 3-aminopropyltriethoxy-silane modified silica xerogel for CO_2_ removal from natural gas. The adsorption capacity of the adsorbent was determined as 1.14 mmol/g at room temperature. Witoon et al.[25] prepared silica xerogel with high silanol content from sodium silicate as a CO_2_ adsorbent with the highest adsorption capacity of 1.90 mmol/g at 35 °C. Echeverría et al. [26] investigated the CO_2_ adsorption performance of ultramicroporous silica xerogels at 0 °C. It was reported that the CO_2_ adsorption capacity of the samples changed between 0.0037 mmol/g and 0.0079 mmol/g depending on the amount of precursors and synthesis temperature.

In this study, polyethylene glycol (PEG)/silica and carbon black (CB)/silica xerogel composites were prepared as an adsorbent for CO_2_ capture for the first time. In order to increase the CO_2_ adsorption capacity, surface modification of the silica xerogel composites was carried out with wet impregnation method using propylene diamine which is quite low-cost compound compared to other amine sources. In contrast to most commonly used amine based wet scrubbing technologies which have some disadvantages (high energy requirement, equipment corrosion, and amine toxicity to the environment), using amine impregnated silica xerogels for CO_2_ adsorption was one of the superiorities of this work regarding environmental and cost aspects. After the structural, morphological, textural, and thermal properties of the silica xerogel composites were investigated by different analyses, CO_2_ adsorption capacity and cyclic stability of the xerogel composites were examined with thermogravimetric analysis (TGA).

## 2. Materials and methods

### 2.1. Materials

Tetraethylorthosilicate (TEOS, Sigma-Aldrich) was used as a silica precursor. Ethanol (EtOH, Merck), nitric acid (HNO_3_, Merck) and ammonium hydroxide (NH_4_OH, Merck) were used as solvent, hydrolysis and condensation catalyst, respectively. In the aging step, isopropanol (Merck) and *n*-hexane (Merck) were utilized as the solvent. Propylene diamine (Sigma-Aldrich) was preferred for the surface modification step. Polyethylene glycol (PEG, Merck) with an average molecular mass of 400 and carbon black (CB, particle size < 45 μm) supplied from Yaroslavskiy Tekhnicheskiy Uglerod were used as filler. All materials were used without any further purification.

### 2.2. Preparation of silica xerogel composites

Silica xerogel was synthesized by the sol-gel method which includes hydrolysis and condensation of TEOS with the molar ratio of TEOS (1.00): H_2_O (4.04): HNO_3_ (2.5 × 10^−3^): NH_4_OH (2.5 × 10^−2^). Firstly, TEOS was dissolved in EtOH, and silica hydrosol was prepared by adding H_2_O and 0.06 M HNO_3_ with stirring for 30 min at room temperature. The silica gel was obtained with 0.1 M NH_4_OH solution dropwise within seconds. After gelation, the silica gel was aged in isopropanol/H_2_O solution (v/v: 1/1) and isopropanol at 50 °C for 1 day, respectively. The gel was washed with *n*-hexane three times during the day. The silica gel was dried at atmospheric pressure at 50 °C for 1 day.

In the preparation of PEG/silica and CB/silica xerogel composites, the silica hydrosols were obtained by adding 10 wt% PEG and 0.33 wt% CB with stirring for 30 min at room temperature before the condensation step, respectively. The condensation reaction was completed with the addition of NH_4_OH solution. The aging and drying steps were applied to silica xerogel composites as mentioned above.

Amine modification of silica xerogel and silica xerogel composites was carried out using the wet impregnation method [27]. Before modification, silica xerogel and xerogel composites were heat-treated in the furnace to remove adsorbed solvents and water molecules. Two grams of each sample was added to propylene diamine/isopropanol solution (w/w: 1/1). The samples were stirred until the formation of semisolid slurry at room temperature. After washing the samples with *n*-hexane, the samples were dried at 50 °C for 1 day at atmospheric pressure.

### 2.3. Characterization of silica xerogel composites

The crystal structure of the samples was investigated using Bruker D8 Advance X-ray diffractometer with Cu-Kα radiation (λ = 1.54 Å) at generator voltage of 40 kV and a generator current of 40 mA with a step size of 0.017° from 10° to 80°. Field emission scanning electron microscopy (FESEM) analysis was performed on TESCAN MAIA3 XMU scanning electron microscope, after coating the sample surfaces with a fine gold layer. Fourier transform infrared spectroscopy (FTIR) analysis was carried out to determine the chemical bonding state of the samples with Bruker Vertex 70 in the range of 4000–400 cm^−1^. Thermogravimetric analysis (TGA) was employed with METTLER STAR SW thermal analyzer at a heating rate of 10°/min from 25 °C to 800 °C. The bulk density of the samples was calculated with the ratio of individual mass of the samples to their volumes. The surface area and pore structure of the samples were investigated with N_2_ adsorption-desorption isotherms using Micromeritics Tristar II 3020 surface area analyzer. The adsorption-desorption isotherms were obtained at 77 K, after the samples were degassed at 473 K for 8 h. The surface area of the samples was determined as per Brunauer–Emmett–Teller (BET) method. The average pore diameter and pore volume of the samples were also specified using Barrett–Joyner–Halende (BJH) method.

### 2.4. CO_2_ capture analysis of silica xerogel composites

The CO_2_ adsorption-desorption measurements of the samples were performed on METTLER STAR SW thermal analyzer as described in the study of Linneen et al. [28]. Initially, about 10 mg of each sample was heated to 100 °C under high purity Ar flow (100 mL/min) at 1 bar for 30 min to remove impurities. The temperature was decreased to 25 °C and pure CO_2_ was inserted into the sample pan at a flow rate of 100 mL/min for 1 h. Moreover, CO_2_ adsorption measurements were also conducted at 25 °C in ambient air. The CO_2_ equilibrium adsorption capacity was calculated with mass gained in the adsorption process.

The samples were heated to 100 °C under high purity Ar flow for 20 min in the first step of CO_2_ adsorption-desorption cyclic stability test. And then, the temperature was decreased to 25 °C and pure CO_2_ was inserted into the sample pan for 20 min. For desorption experiments, Ar gas was inserted into the sample pan at 100 °C for 20 min again. The stability test was repeated five times for each sample.

## 3. Results

### 3.1. XRD analysis

**Figure 1:**
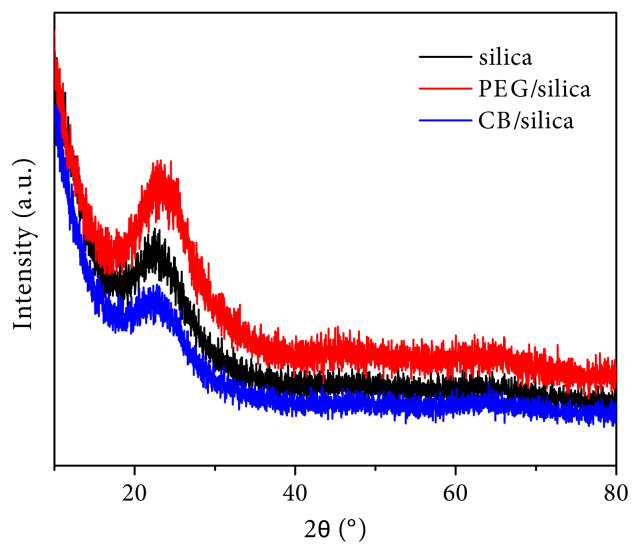
XRD patterns of the samples.

The XRD patterns of the samples are shown in Figure 1. It was clearly seen that there was no obvious diffraction peak related to crystallinity and the typical broad peak at 2θ = 23° was an indication of the amorphous structure of SiO_2_ [29].

### 3.2. FESEM analysis

**Figure 2:**
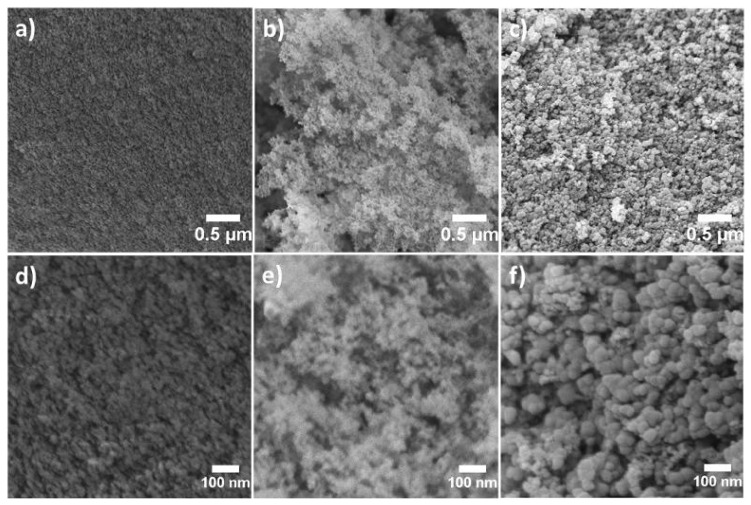
FESEM image of **a)** silica xerogel, **b)** PEG/silica xerogel composite, **c)** CB/silica xerogel composite, high magnification of FESEM image of **d)** silica xerogel, **e)** PEG/silica xerogel composite, and **f)** CB/silica xerogel composite.

The FESEM images of the samples are shown in Figure 2. The porous structure of the silica xerogel was not clearly observed because of its smaller pore size (Figures 2a and 2d) that may originate from irreversible shrinkage of silica gels in the drying step. Compared to silica xerogel, silica xerogel composites had more porous network. PEG/silica xerogel composite exhibited uniform reticular structure related to the linear molecular structure of PEG molecules (Figures 2b and 2e) [30]. Pearl-necklace morphology was seen in the FESEM image of the CB/silica xerogel composite due to the spherical shape of CB particles (Figures 2c and 2f). In other words, the formation of an interlinked silica network was promoted on the surface of CB particles [31].

### 3.3. FTIR analysis

**Figure 3:**
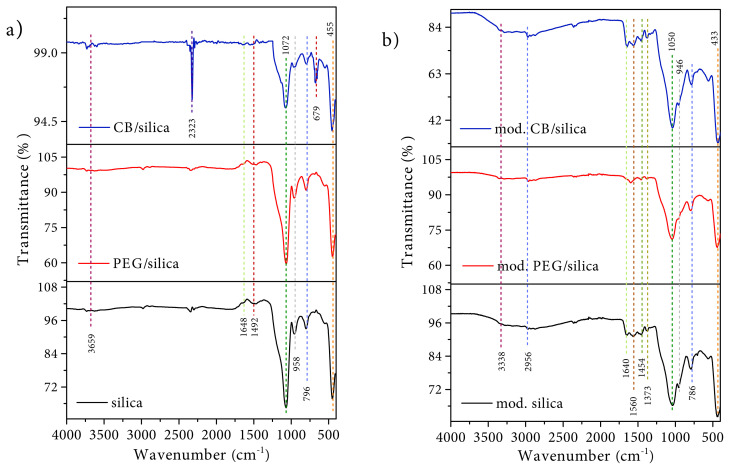
FTIR spectra of **a)** pure and **b)** amine modified samples.

The FTIR spectra of pure and modified samples are shown in Figure 3. The characteristic peak belonging to Si-O-Si bond appeared at around 1072 cm^−1^ due to asymmetric stretching vibrations for all samples (Figure 3a) [32]. The peaks at 455 cm^−1^ and 796 cm^−1^ corresponded to the bending of O-Si-O bond and symmetric bending of Si-O-Si bond, respectively [33]. The stretching vibrations of Si-OH and Si-O^−^ were observed at around 958 cm^−1^[34]. As well as the band at about 3659 cm^−1^, the peak at 1648 cm^−1^ was attributed to −OH stretching vibrations related to adsorbed water molecules which provide surface modification easily. The peak originated from C-H vibrations was determined at 1492 cm^−1^[35]. Moreover, the peaks at 679 cm^−1^ and 2323 cm^−1^ indicated Si-C stretching vibrations and CO_2_ in CB/silica xerogel composite, respectively [36].

After modification, the symmetric and asymmetric bending vibrations of NH_2_ groups were seen at 1454 cm^−1^ and 1560 cm^−1^ (Figure 3b). The peaks at 1373 cm^−1^ and 2956 cm^−1^ were attributed to the characteristic CH_2_ stretching mode of amine chains [37]. Additionally, the characteristic peaks of the silica network slightly shifted to the right through amine-based wet impregnation process [38]. The presence of amine and hydroxyl groups in FTIR spectra demonstrated that the silica xerogel composites can be available for CO_2_ adsorption by interactions between these groups and CO_2_ molecules [39].

### 3.4. TGA analysis

**Figure 4:**
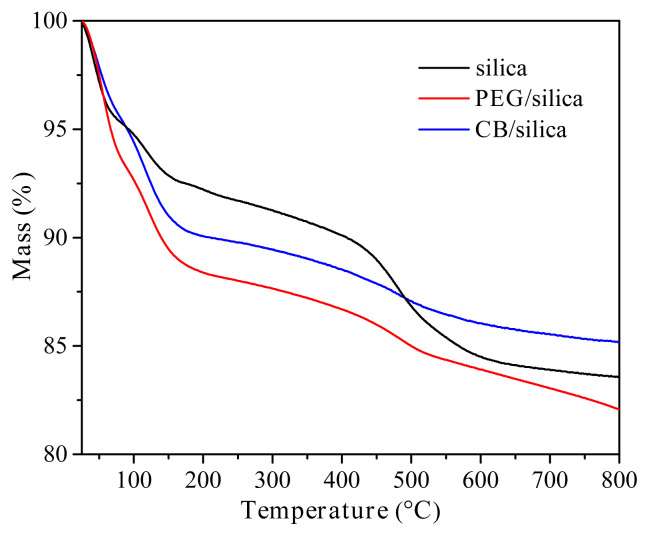
Mass of the samples as a function of temperature.

The mass of the samples as a function of temperature is shown in Figure 4. It was clearly seen that the residue of all samples at 800 °C was higher than 80%, which revealed the good thermal stability of the samples. The initial mass loss originated from the evaporation of residual adsorbed solvents and water from the samples. An increase in temperature from 200 °C to 500 °C caused a mass loss depending on the decomposition of residual organic groups [40]. PEG/silica xerogel composite showed lower thermal resistance compared to other samples. It can be the result of decomposition of the pure PEG molecules in the range of 200–400 °C, so this induces a decrease in thermal resistance of the sample [41]. Above 500 °C, the thermal decomposition of the samples also continued until the removal of organic structures from the samples.

### 3.5. BET analysis

**Figure 5:**
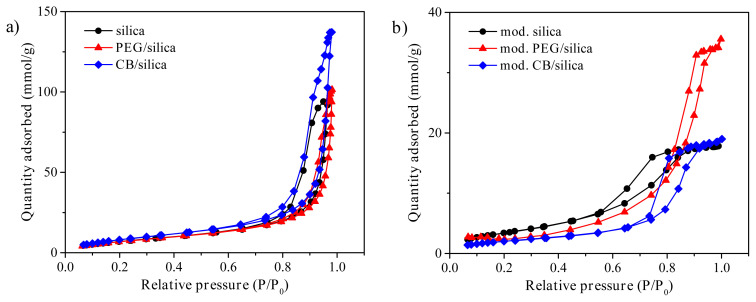
N_2_ adsorption-desorption isotherms of **a)** pure and **b)** amine modified samples.

The N_2_ adsorption-desorption isotherms of the samples are shown in Figure 5. The specific surface area and pore structure properties of the samples are also given in detail in Table. It was observed that all samples had type-IV isotherm which is the distinctive feature of mesoporous materials [42]. According to IUPAC classification, the pores are classified as ultramicroporous (below 0.8 nm), microporous (between 0.8 and 2.0 nm), mesoporous (between 2.0 and 50.0 nm) and macroporous (above 50.0 nm) [43]. The average pore size of the samples changed between 6 nm and 17 nm. This result exhibited that mesoporous materials were prepared in this study. Incorporation of small amount of PEG and CB to the silica structure provided to increase its specific surface area, pore volume, and average pore diameter. It is well known that a certain amount of porogen materials like PEG and CB can easily enhance the textural properties of the samples [30]. Similar results were reported for different purposes in the literature [44,45].

The modification process significantly decreased the surface area, pore volume, and average pore diameter of the samples, which can be explained with an additional shrinkage process [37]. The combination of capillary force with weak integrity of the samples causes the shrinkage in the ambient pressure drying step. Moreover, the type of modification agents such as mono-, di- and tri-amines considerably affects the surface area and pore structure of the samples [46].

As can be seen from Table, the bulk density of the pure silica xerogel showed a slight increase to 0.184 g/cm^3^ and 0.194 g/cm^3^ in the presence of PEG and CB before modification, respectively. In spite of the higher pore volume of the xerogel composites, the individual density of the PEG or CB was dominant in the determination of bulk density [30]. Obviously, the bulk density of the samples dramatically increased in case of the modification process. The addition of modification agents generally increases the mass of the samples, while it has a slight effect on the volume. So, an increase in the bulk density is expected as such in many studies in the literature [47,48].

### 3.6. CO_2_ capture analysis

**Figure 6:**
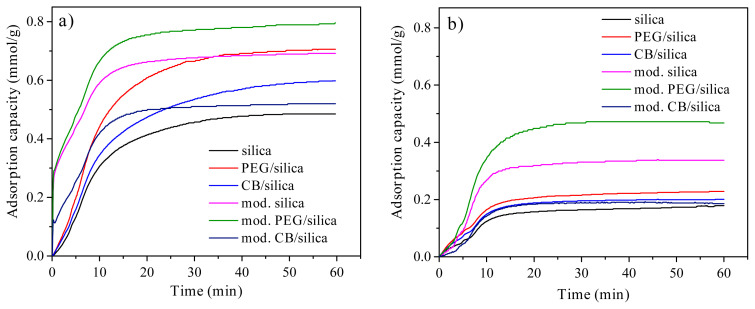
CO_2_ adsorption capacities of the samples **a)** under pure CO_2_ exposure and **b)** in ambient air.

The CO_2_ adsorption capacities of the samples as a function of time are shown in Figure 6. It was possible to divide the adsorption process into two stages under pure CO_2_ exposure (Figure 6a). While the CO_2_ adsorption capacity of the samples sharply increased in the first stage, the adsorption rate decreased in the second stage. It was related to decreasing available sites in time for the CO_2_ capture [27]. It was clear that unmodified samples reached 90% of their equilibrium adsorption capacities after 25 min of CO_2_ exposure. The equilibrium adsorption capacity was determined as approximately 0.48 mmol/g for pure silica xerogel. The incorporation of PEG and CB increased the equilibrium adsorption capacity to 0.70 mmol/g and 0.59 mmol/g, respectively. It can be explained with larger pore size and volume of the samples which reduce mass transfer resistance of CO_2_ promoting an increase in adsorption capacity [49]. In the literature, the positive effect of a certain amount of PEG on the CO_2_ adsorption performance was reported for fumed silica [50]. Another case to consider was that PEG/silica xerogel composite showed higher adsorption capacity than CB/silica xerogel composite. It can be resulted from the addition of CB into the silica sol before the condensation step, which may lead to an undesirable interaction between CB and HNO_3_ catalyst. A similar trend was observed for the adsorption performance of the samples in ambient air as shown in Figure 6b. In a short time, CO_2_ adsorption equilibrium was reached for all samples. Compared to adsorption capacity under pure CO_2_ exposure, CO_2_ adsorption capacity of the samples decreased due to the low concentration of CO_2_ in ambient air as expected such in literature studies [51]. CO_2_ adsorption capacity of pure silica xerogel was about 0.18 mmol/g. For PEG/silica and CB/silica xerogel composites, CO_2_ equilibrium adsorption capacity was determined as 0.23mmol/g and 0.20 mmol/g, respectively.

After amine modification, the samples reached equilibrium adsorption capacity within the first 10 min (Figure 6a). In spite of a decrease in surface area and pore volume of the samples with the modification process, the amine based wet impregnation method provided more available binding sites for CO_2_ molecules, and therefore CO_2_ adsorption rate was effectively increased. Amine groups depending on loading capacity and nitrogen content generally assist the CO_2_ capture through interactions between these groups and CO_2_ molecules by hydrogen bonding. In the absence of water, the adsorption process is performed by a zwitterionic mechanism to form carbamates with a stoichiometric ratio of 2 mol of N to 1 mol of CO_2_ [52]. It was obvious that modified samples had higher equilibrium adsorption capacity than unmodified samples except for CB/silica xerogel composite. The maximum adsorption capacity was specified as about 0.80 mmol/g for amine modified PEG/silica xerogel composite. The equilibrium adsorption capacity of CB/silica xerogel composite slightly decreased from 0.59 mmol/g to 0.52 mmol/g with the amine modification. The amine impregnation did not seem to be a useful method for CB/silica xerogel at room temperature to enhance CO_2_ adsorption capacity in contrast to other xerogel composites. For modified silica xerogel and PEG/silica xerogel composite, it was clear that CO_2_ adsorption capacity increased by the synergistic effects of amine groups in spite of a decrease in their surface area and pore volume. However, as well as deterioration in textural properties of CB/silica xerogel composite, amine impregnation affected the adsorption capacity of the sample negatively. This result was in agreement with that of the many CO_2_ adsorption studies including carbon-based materials in the literature [53]. It can be expressed by the isosteric heat of CO_2_ adsorption which indicates the strength of the interaction between adsorbate and adsorbent. CO_2_ adsorption capacity significantly depends on pore structure at low pressure, while amine modification has no critical influence. And also, instead of low temperatures, amine modification at medium temperatures can be more influential for CO_2_ chemisorption of these types of materials [54,55].

CO_2_ capture analysis in ambient air represented that the CO_2_ adsorption performance of the modified samples was better than that of the unmodified samples (Figure 6b). Amine modified PEG/silica xerogel composite showed maximum CO_2_ adsorption capacity approximately 0.43 mmol/g in ambient air. However, the CO_2_ capture process in ambient air led to lower adsorption capacity than dry 100% CO_2_exposure that can be attributed to a decrease in functionality of amine groups in case of O_2_ contact as well as presence of humidity and considerably low CO_2_ concentration. For example, water molecules in the air can behave as a competitive adsorbate against CO_2_ molecules that generally reduce CO_2_ adsorption capacity of the materials [56].

CO_2_ adsorption capacity of many amine modified mesoporous silica-based materials changes between 0.41 mmol/g and 5.9 mmol/g depending on type of amine-based agents and modification method, temperature and pressure under pure CO_2_ atmosphere in the literature [2]. In spite of lower CO_2_ adsorption capacity of the materials in this study, silica xerogel composites still have advantages such as simple synthesis process, tunable pore structure, easy surface modification or coating due to their hydrophilic structure, and ability of rapid CO_2_ capture. Further development including change in the synthesis parameters of silica xerogel composites, using another amine-based modification agents rich in nitrogen content, altering modification and adsorption conditions can be considered to enhance CO_2_ adsorption capacity of the materials.

### 3.7. Regenerability of adsorbents

**Figure 7:**
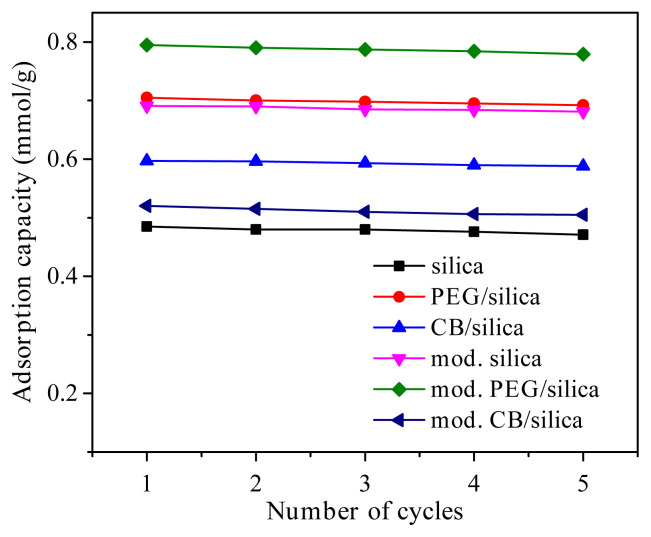
CO_2_ adsorption-desorption cyclic stability of the samples

The long-term stability of the adsorbents is an important factor in addition to high CO_2_ adsorption capacity for many adsorption applications. The cyclic performance of the samples is shown in Figure 7. It was reported that excellent stability was obtained during the five cycles for all samples. The CO_2_ adsorption capacity of the samples almost remained constant, which is generally observed for many effective CO_2_ adsorbents [57,58]. Easy and time effective regeneration of the materials can be regarded as the promising property of the silica xerogel composites.

## 4. Discussion

The mesoporous PEG/silica and CB/silica xerogel composites were successfully prepared as CO_2_ adsorbent with a simple method at ambient pressure. The xerogel composites showed higher surface area and pore volume compared to pure silica xerogel, which increased equilibrium CO_2_ adsorption capacity. After amine modification, an increase in the adsorption capacity of the samples was determined in spite of a decrease in surface area and pore volume of the samples. The highest equilibrium adsorption capacity was obtained as about 0.80 mmol/g for amine modified PEG/silica xerogel composite under pure CO_2_ exposure at 25 °C and 1 bar. However, amine modification adversely affected the CO_2_ adsorption capacity of CB/silica xerogel composite. Moreover, all samples exhibited stable performance in the CO_2_ adsorption-desorption cyclic stability test.

Nowadays, the usage of silica xerogels as CO_2_ adsorbent is the newly-emerging research subject. Even now, the adsorption capacity of the various pure silica xerogels is lower at ambient conditions than that of the other CO_2_ capture technologies. However, the technologies have many disadvantages such as corrosion, toxicity, complexity, the necessity of high energy, high-cost and so on. And, silica xerogel composites still have advantages of flexible synthesis method, tunable textural properties, their hydrophilic structure, and ability of rapid CO_2_ adsorption. Therefore, exhaustive investigations should be performed to improve the adsorption capacity of the silica xerogel composites.

## Figures and Tables

**Table:** The bulk density and BET analysis results of the samples.

Sample	Bulk density (g/cm^3^)	BET surface area (m^2^/g)	Pore volume (cm^3^/g)	Average pore diameter (nm)
silica	0.144	594	1.64	10.29
PEG/silica	0.184	756	2.66	15.78
CB/silica	0.194	798	3.21	17.25
mod. silica	0.496	293	0.63	6.72
mod. PEG/silica	0.516	171	1.23	12.48
mod. CB/silica	0.713	174	0.64	12.05
